# Exploring the Potential of Machine Learning for the Diagnosis of Balance Disorders Based on Centre of Pressure Analyses

**DOI:** 10.3390/s22239200

**Published:** 2022-11-26

**Authors:** Fredy Rojas, Imran Khan Niazi, Patricio Maturana-Russel, Denise Taylor

**Affiliations:** 1 Department of Mathematical Sciences, Auckland University of Technology, Auckland 1010, New Zealand; 2 Centre for Chiropractic Research, New Zealand College of Chiropractic, Auckland 1060, New Zealand; 3 Health and Rehabilitation Research Institute, Auckland University of Technology, Auckland 1010, New Zealand; 4 Center for Sensory-Motor Interaction, Department of Health Science and Technology, Aalborg University, 9220 Aalborg, Denmark; 5 New Zealand Dizziness and Balance Centre, Auckland 0627, New Zealand

**Keywords:** balance disorder, approximate entropy, empirical mode decomposition, machine learning model

## Abstract

Balance disorders are caused by several factors related to functionality deficits in one or multiple sensory systems such as vision, vestibular, and somatosensory systems. Patients usually have difficulty explaining their dizziness, often using ambiguous words to describe their symptoms. A common practice by clinicians is to objectively evaluate the patient’s dizziness by applying the Sensory Organization Test (SOT), which measures the contribution of each sensory system (vestibular, visual, somatosensory). The SOT protocol can record up to 2000 measurements in 20 s to generate the Equilibrium Score (EQS) with its five load sensors. EQS is an indicator that reflects how well a patient can maintain balance. However, its calculation only considers two instances from these 2000 measurements that reflect the maximum anterior and posterior sway angle during the test performance; therefore, there is an opportunity to perform further analysis. This article aims to use the Centre of Pressure (COP) time series generated by the SOT and describes a methodology to pre-process and reduce the dimensionality of this raw data and use it as an input for machine learning algorithms to diagnose patients with balance disorder impairments. After applying this methodology to data from 475 patients, the logistic regression model (LR) produced the highest f1-score with 76.47%, and the support vector machine (SVM) performed almost as well, with an f1-score of 76.19%.

## 1. Introduction

Balance is crucial for an individual’s mobility and physical independence and is commonly impacted with ageing [1]. The human balance system is a complex system of sensory organs and mechanisms, including vestibular, visual, and somatosensory inputs. The constant flow of information from those inputs, as well as a complex array of motor outputs [2] is processed in the brain to generate the sensory inputs controlling the eye, head, neck, trunk, and leg position to maintain body equilibrium.

Diagnosing the root cause of dizziness can be difficult for clinicians due to subjective symptom explanations that cannot be measured and the wide range of health conditions that these symptoms can be related to [3]. Dizziness is commonly used to describe a range of sensations, such as vertigo, light-headedness, faintness, and imbalance. Patients often find it hard to explain their dizziness symptoms and use ambiguous terms that could involve several possible causes. Therefore, a wide range of root causes and imprecise dizziness symptom descriptions make it difficult for clinicians to assess dizziness and select optimum treatments.

**Figure 1:**
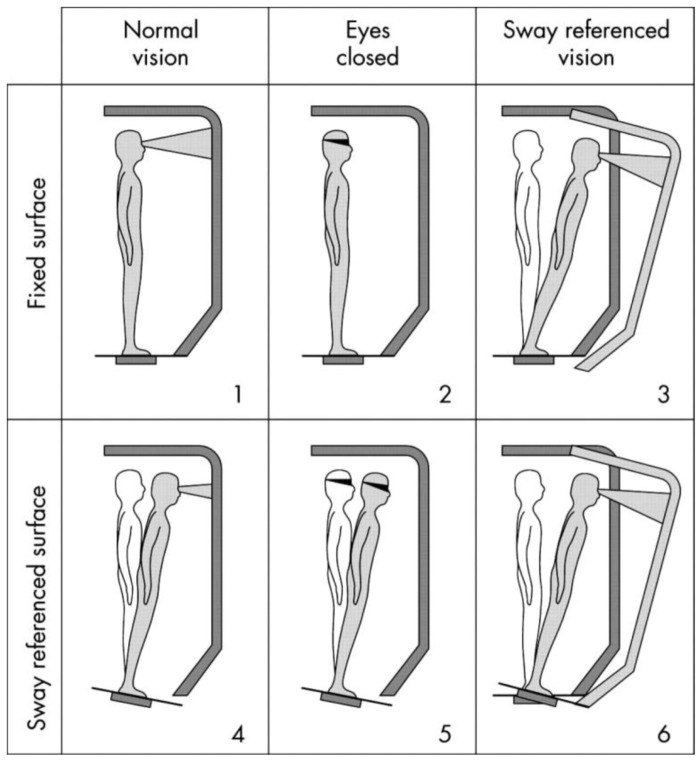
The 6 conditions of the Sensory Organization Test (SOT) constitute the following: (**1**) eyes open, stable support; (**2**) eyes closed, stable support; (**3**) sway-referenced vision, stable support; (**4**) eyes open, sway-referenced support; (**5**) eyes closed, sway-referenced support; and (**6**) eyes open, sway-referenced vision, and sway-referenced support [6].

Computerised Dynamic Posturography (CDP) evaluates the contribution of each sensory system (vestibular, visual, somatosensory) to maintaining body equilibrium under static or dynamic conditions. CDP protocols aim to detect abnormalities in the use of the sensory systems and the most commonly used protocol is the Sensory Organization Test (SOT) [4]. The principle of the SOT is to create a selective disruption in the support surface, the visual surroundings, or both, to measure a person’s ability to use the remaining sensory inputs to keep body balanced [5]. As is shown in Figure 1, the SOT has six test conditions. The difficulty of each condition gradually increases from condition one up to condition six. In the first condition, everything is steady, and in the sixth condition, everything is non-fixed (visual surroundings and support plate).

The Smart Equitest (Neurocom/Natus) Computerised Dynamic Posturography can perform SOT evaluation. This machine can control the movement of the visual surroundings and force plate, and has a harness to ensure patient safety throughout the testing protocol. As part of the diagnostic assessment, the Smart Equitest generates a standard comprehensive report that includes the Equilibrium Scores (EQS) of the six test conditions, four ratios of sensory analysis, results of strategy analysis, and information about the Center of Gravity (COG) alignment. The four ratios of sensory analysis are Somatosensory (SOM), Visual (VIS), Vestibular (VEST), and Visual Preference (PREF). In conjunction with EQS, these results help to identify abnormal contributions in the use of an individual’s sensory systems [7].

The EQS is an indicator that reflects overall balance with a single value between 0 and 100 for each SOT condition. According to Zammit (2008) [8], an EQS of 100 represents perfect balance (no sway), and an EQS of 0 represents when sway exceeds the theoretical stability limits. EQS is calculated according to a simple formula:

(1) $$\mathrm{EQS}\;=\frac{12.5- [\theta_{\max}\left(\mathrm{ant}\right)-{\;\theta }_{\max}\left(\mathrm{post}\right)]}{12.5}$$

where θ_max_(ant) is the maximum anterior sway angle in degrees during the test conditions, θ_max_(post) represents the posterior sway angle in degrees for the same test conditions, 12.5 is the limit of sway in degrees in the sagittal plane in a normal stance, and 12.5° is assumed to be the limit of stability for a normal individual (approximately 7° anteriorly and 5° posteriorly [9]). However, according to Chaudhry et al. (2011) [10] there are some ambiguities/disadvantages that should be considered while using EQS:

- The stability limits may vary significantly by age and height.
- There is an asymmetry in the average value representing the limit of stability for normal balance participants (approximately 7° anterior and 5° posterior sway) that is disregarded in the EQS equation.
- More than one combination of anterior and posterior sway degrees can result in the same EQS value.
- The EQS only considers the two extreme values of the sway angle in a given test condition, not the complete measurement history (2000 data points) in a trial of 20 s.

The CDP machine is able to measure the centre of pressure (COP) at a rate of 100 Hz from the load cells located in the support plate during the performance of the SOT conditions. The COP is the sum of all the pressure forces over the CPD platform generated by the patient during the SOT conditions. These COP measurements are part of the SOT raw data, and the CDP machine uses them to generate the EQS. The COP has demonstrated clinical utility as an indicator of postural control performance [11,12]. According to Cavanaugh and Guskiewic (2005) [12], the COP’s erratic appearance contains a hidden structure, or orderliness, that emerges over time due to the interactions among underlying postural control system components. Therefore, considering the ambiguities mentioned by Chaudhry et al. (2011) [10], it was decided to use the raw COP data (2000 points) from SOT conditions in this study.

It is essential to consider that coordinated human movement comprises the integration of multiple degrees of freedom (e.g., motor unit, muscle, joint) into coherent functional units. In research on human movement, it is widely accepted that there is a redundancy in the degrees of freedom that allows our control system to generate different solutions for the same task [13]. Additionally, a growing body of literature expresses that postural stability is achieved through the interaction of various systems. Therefore, resulting postural control measurement techniques may be naturally nonlinear and thus might be best studied via analyses based on nonlinear dynamical approaches [14].

According to Ivanenko and Gurfinkel (2018) [15], the postural control system in humans is characterised by non-linear behavior. In this situation, one of the most popular tools used in human signal analysis is entropy. Entropy quantifies the regularity of nonlinear dynamics systems. Thus, the more regular a series, the more predictable and less complex it will be, indicating a less adaptive system. Over time, the mathematical methods to calculate entropy have evolved from Approximate Entropy (ApEn) up to the Multi-scale Entropy (MSE) [16].

The SOT protocol measures the contribution of each sensory system to the maintenance of balance, but it does not classify or diagnose patients. Previous studies have used COP, Empirical Mode Decomposition (EMD), or ApEn to create new indices to enhance the performance of the SOT protocol in measuring the sensory system contributions or to find significant effects of illnesses over the SOT conditions using these new indices [4,6,7,9,10]. Keeping in mind that the EQS is calculated from 2 points out of the 2000 points recorded by the CDP machine, we considered that there is an opportunity to obtain more information from this data. Therefore, this study aims to investigate whether a machine learning approach can expand the usability of the SOT raw data to classify patients into particular balance impairment groups. For that, we proposed a feature-based method using the ApEn algorithm, representing each COP sway trajectory with a single value, thus reducing the input data dimensionality and improving machine learning performances. The rest of this paper is structured as follows. Section 2 introduces the participants’ description and definition of the main methods used in this article. Section 3 describes the characteristics of the reduced dataset after applying ApEn and the resulting predictive power of the machine learning model. Section 4 describes observations from Section 3 and Section 5 formulates conclusions from the previous analysis.

## 2. Materials and Methods

### 2.1. Subjects

**Figure 2:**
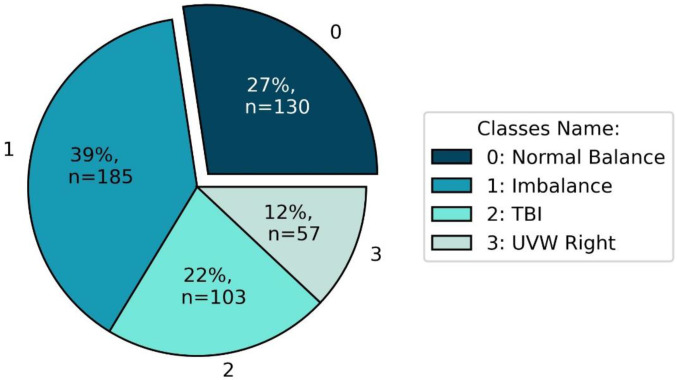
Percentage distribution of subjects per diagnosis.

The four most frequent balance disorder diagnoses were selected from anonymised patient records collected from 2012 to 2021. These diagnoses are Normal Balance, Imbalance, Traumatic Brain Injury (TBI), and Unilateral Vestibular Weakness Right (UVW Right). Figure 2 shows the number of patients and proportion of the selected balance disorder impairments. The Imbalance diagnosis group is the largest, with 39% of the total number of patients, while, with 12%, the UVW Right is the smallest group. In more detail, Figure 2 shows that the Normal Balance group has 130 individuals (mean age 46.30 ± 14.27, range age: 8–84), the Imbalance group has 185 patients (mean age 56.84 ± 19.48, range age: 6–89), the TBI group has 103 patients (mean age 48.51 ± 15.71, range age: 7–81), and the UVW Right group has 57 patients (mean age 61.19 ± 13.17, range age: 26–85) making a total of 475 patients. Globally the patients’ ages ranged between 6 and 89 years old (age 52.7 ± 17.5 years; height 169.3 ± 10.4 cm.). The data used in this study come from the SOT performance, which is part of the patients’ balance disorder diagnosis process.

### 2.2. Approximate Entropy (ApEn)

ApEn evaluates the amount of randomness in each test condition of collected COP data. The ApEn algorithm calculates the probability that a short sequence of consecutive data points repeats throughout a more extended temporal series of points, expressing the average probability in logarithmic form. ApEn generates a single value that represents how random (or predictable) a time series is. An ApEn with value zero corresponds to a time series in which the sequences of data points are perfectly repeatable (e.g., a sine wave) [17,18]. A consideration mentioned by Pincus and Goldberger (1994) [19] is that trending on time series will spuriously lower the ApEn estimates. Therefore, from a statistical perspective, it is necessary to eliminate any trend before making meaningful interpretations from the ApEn algorithm results.

In more detail, Pincus and Goldberger (1994) [19] proposed the algorithm to calculate ApEn as follows, given N data points u(1), u(2), ... u(N), two parameters must be fixed m (embedding dimension) and r (comparison tolerance). After that, we define the blocks by x(i) = [u(i), ..., u(i + m − 1)] and x(j) = [u(j), ..., u(j + m − 1)] and calculate the distance between them as d[x(i), x(j)]. Then we calculate the value $C_{i}^{m}\left(r\right)$ given by

(2) $$C_{i}^{m}\left(r\right)=\frac{\left(\mathrm{no}.\;\mathrm{of}\;j\;\leq \;N\;-\;m+1\;\mathrm{such}\;\mathrm{that}\;d [x\left(i\right),\;x\left(j\right)]\leq \;r\right)\;}{\left(N\;-\;m\;+1\right)}$$

The $C_{i}^{m}\left(r\right)$ measure, within a tolerance, r the regularity, or frequency, of patterns similar to a given pattern of length m. With Equation (2), the ApEn is given by

(3) $$\mathrm{ApEn}\left(m,\;r,\;N\right)=\frac{1}{\left(N-m+1\right)}\overset{N-m+1}{\underset{i-1}{\sum }}{\log\;C}_{i}^{m}\left(r\right)-\frac{1}{\left(N-m\right)}\overset{N-m}{\underset{i-1}{\sum }}{\log\;C}_{i}^{m+1}\left(r\right)$$

The ApEn calculations are performed with the Python Library Antropy.

### 2.3. Empirical Mode Decomposition (EMD)

According to Gow et al. (2015) [20], the Empirical Mode Decomposition (EMD) as a filter bank is the technique most commonly used to overcome the effect of nonstationary data. The EMD method was specially developed for decomposing non-linear, non-stationary signals into their intrinsic mode functions (IMFs). Unlike Fourier and wavelet methods, there are no a priori assumptions about the nature of the signal, and it does not rely on a specific basis (e.g., sinusoidal or Haar wavelet function) to decompose the signal. After decomposition by EMD, the resulting IMFs can be recombined in different ways, representing a range of characteristics of the original signal [21]. It is expected that the trend of the original signal is captured by the IMFs with lower frequencies. Therefore, by subtracting them, the process of detrending can be achieved. As a rule of thumb, Costa et al. (2007) [22] combine the five highest IMF frequencies to be analysed by techniques such as ApEn.

The algorithm to apply EMD can be summarised as follow:

1. Identify all extrema of the signal x(t).
2. Fit the maxima and minima to an individual envelope $e_{\mathrm{up}}\left(t\right)$ and $e_{\mathrm{low}}\left(t\right)$.
3. Compute the average:
  (4) $$m\left(t\right)=\frac{e_{\mathrm{up}}\left(t\right)+{\;e}_{\mathrm{low}}\left(t\right)}{2}$$
4. Extract the detail:
  (5) $$d\left(t\right)=\;x\left(t\right)-\;m\left(t\right)$$
5. Check the stopping criterion:
  (6) $$\sum_{t}\frac{{\left(d\left(t\right)-\;x\left(t\right)\right)}^{2}}{d^{2}\left(t\right)}<\varepsilon$$
6. If d(t) does not satisfy the stopping criterion, another iteration from steps 1 to 5 using d(t) in step 1 is undertaken until the stopping criterion is fulfilled.
7. When the stopping criterion is fulfilled, only then is the d(t) considered as an IFM. After that, the original x(t) is updated by subtracting the IFM, and the loop starts again at step 1.
8. The decomposition stops when d(t) approaches a monotonic function where is it not possible to extract any extrema.

This study performed the EMD calculation using the Python Library PyEMD.

### 2.4. Machine Learning Methods

According to Molnar (2020) [23], machine learning is a set of methods that allows computers to learn from data to make and improve predictions. Machine learning is a shift from “normal programming” where a programmer gives all the instructions to the computer to “indirect programming,” where the algorithm itself creates its own rules (instructions) directly from the data. Some of the supervised machine learning techniques will be used in this project and are briefly described in the following lines:

- Random Forest (RF) is a general purpose regression and classification machine learning algorithm. Its approach generates several randomised decision trees and aggregates their votes for a final prediction. RF has shown good performance in datasets where the dimensional feature space is greater than the number of observations [24].
- Linear Discriminant Analysis (LDA) is a technique for data classification and dimensionality reduction. It works by maximising the distances between the means of the categories and minimising the variability within them. After fitting the training data, the method generates a linear decision boundary to classify unlabelled observations [25].
- Support Vector Machine (SVM) is an algorithm that looks for a particular line or decision boundary, termed hyperplane, which efficiently separates classes and avoids extra overfitting. This decision boundary is created using a soft margin which is a method that allows misclassification. After fitting the data, the algorithm arranges the hyperplane in such a way that results in better predictions. SVM is capable of performing linear and non-linear classification. For non-linear classification, SVM uses a Kernel function that helps to map the data to high dimensional space. This allows SVM to create non-linear boundaries for classifications [26].
- Logistic Regression (LR), regardless of its name, is a linear model for classification rather than regression. It has its basis in taking the natural logarithm of the odds as a regression function of the predictors. LR can handle both binary and multiclass classification. Unlike statistics approaches, in the machine learning, this approach commonly applies regularisation methods to avoid overfitting [27,28].

### 2.5. COP Time Series Pre-Processing

**Figure 3:**
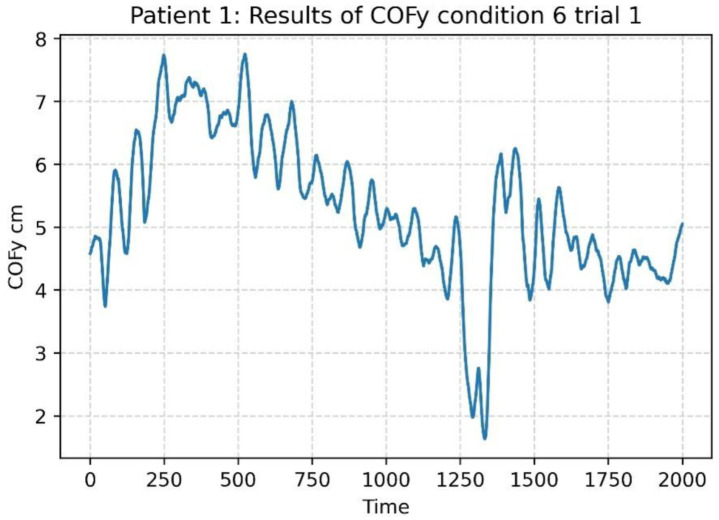
COPy time series for a patient for the test condition 6.

The COP data consist of 2000 observations over time for each patient. It is not unusual that the data sets have a trend; as an example, Figure 3 shows the COP time series of one patient. According to Pincus and Goldberger (1994) [19], trending on the time series underestimates the ApEn values; therefore, Empirical Mode Decomposition (EMD) was applied as a filter bank to remove the trend from the COP data for each test condition of each patient.

**Figure 4:**
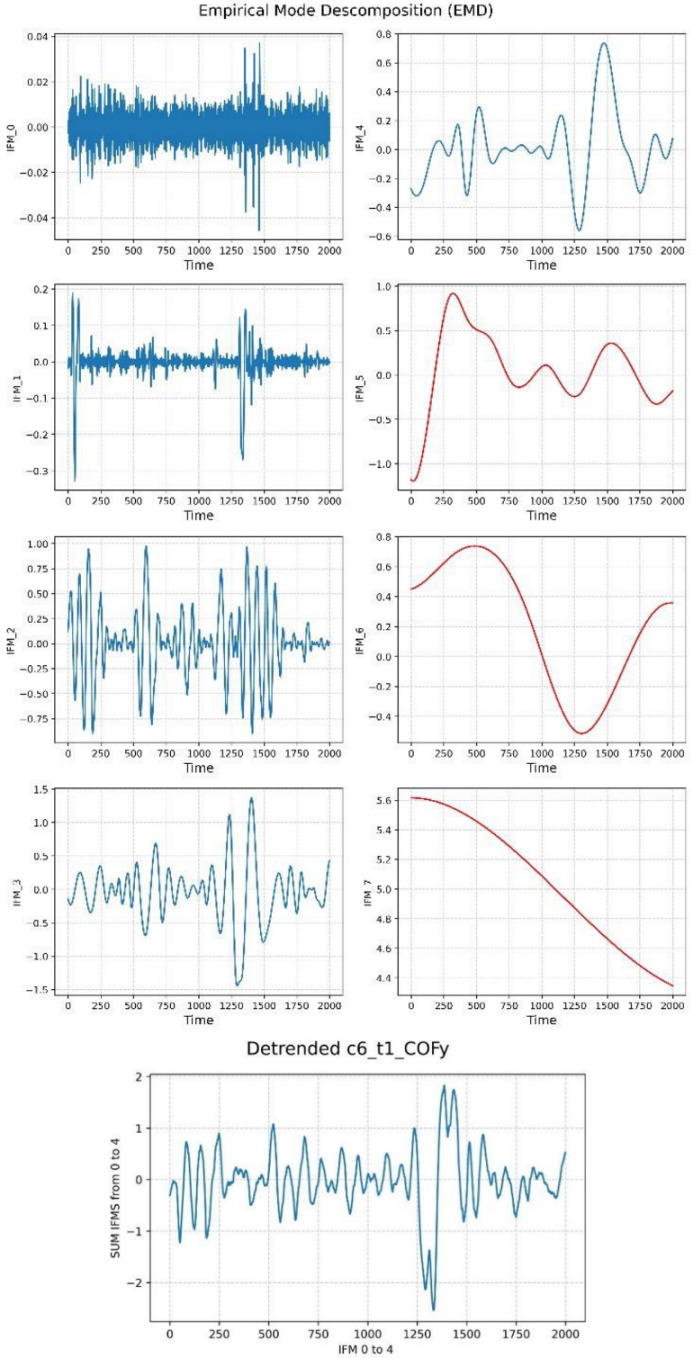
Empirical Model Decomposition to detrend COP.

EMD decomposes the COP time series into IMFs. Detrending the COP time series is possible by extracting and adding the first five IMFs with the highest frequencies [22]. Figure 4 shows the detrending process using EMD. The plots on the top side represent the decomposition of the COPy presented in Figure 3 into eight IMFs. As we can see, the first IMFs captured the higher frequencies of the COP time series, and this gradually decreases up to the eighth IMF, which means that the trend is captured by the last IMFs. This order allows application of the next step, detrending the COP by only adding the first five IMFs. This result can be seen at the bottom of the plot. Given that there are six test conditions of SOT with two COP components, one time series for the axis *x* and the other for the axis *y*, each patient ends up with 12 detrended COP time series.

**Figure 5:**
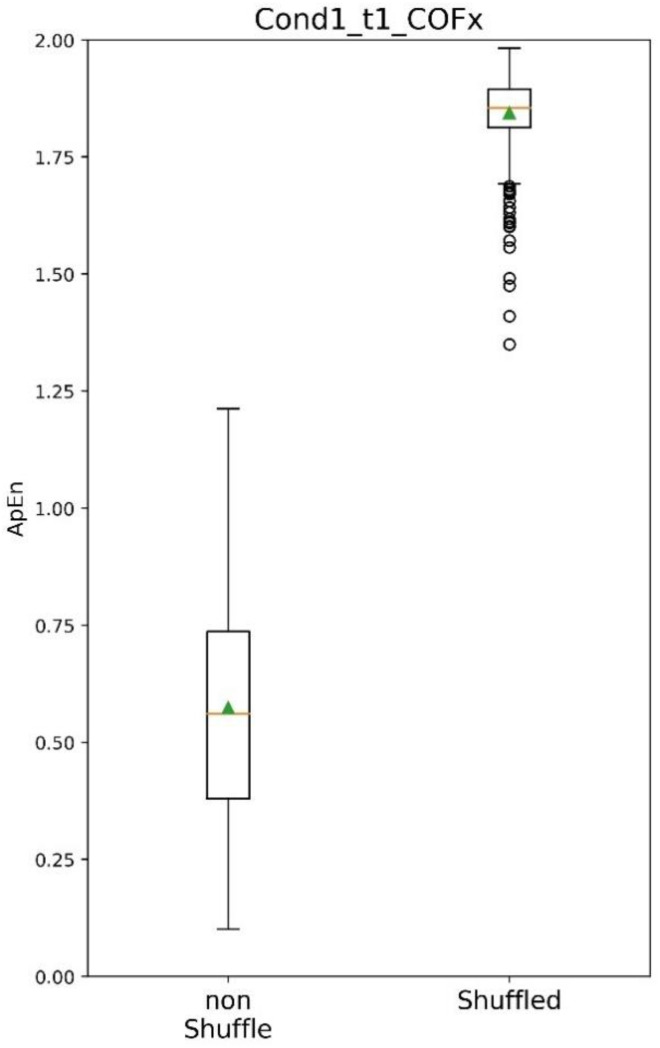
Shuffled/non-shuffled COP—Testing its randomness.

COP is characterised by an erratic appearance. To ensure that the time series still preserved its dependency structure, the complexity (ApEn) of the original detrended COP time series was compared with a shuffled version of itself. Figure 5 shows the results of this comparison, and there is a clear difference between the detrended COP time series complexity and the randomised COP time series on SOT condition 1. This behaviour is true for all of the SOT conditions.

After applying EMD as a detrending method, ApEn was applied to the detrended COP time series. As a result, we obtained 12 unique values of ApEn per patient, which will be used in the next section.

### 2.6. Testing Normality of ApEn Values

ApEn values are the only feature that will be used as the input for machine learning algorithms to assess if it is possible to expand the usage of the SOT raw data. As there are four classes, we will take two approaches. First, we will train the model with the two classes with higher differences. Secondly, we will train the model to classify patients into four classes. To achieve the first approach, it is necessary to determine if parametric tests can be applied to test differences between classes. Therefore, the Shapiro–Wilk test was applied. The Shapiro–Wilk test is a frequentist statistical method that calculates the W statistic to assess whether the sample data comes from a normally distributed population. The W test is given by

(7) $$W\;=\frac{{\left(\sum_{i=1}^{n}a_{i}x_{\left(i\right)}\right)}^{2}}{\sum_{i=1}^{n}{\left(x_{i}-\;\bar{x}\right)}^{2}}$$

where $x_{i}$ are the ordered random values and $a_{i}$ are constants generated from the means, covariances, and variances sampled from the standard normal distribution [29]. This study used the scipy.stat.shapiro Python Library to perform all the calculations of the Shapiro–Wilk test for normality.

### 2.7. Finding the Two Classes with Significant Differences

The Two-Sample Kolmogorov–Smirnov test allows us to compare two samples and tells us whether both samples were drawn from the same (but unknown) distribution. This test evaluates the greatest distance between the cumulative distribution function (CDFs) of each sample using the statistic D that is given by

(8) $$D_{m,\;n}=\underset{x}{\max}\left|F\left(x\right)-\;G\left(x\right)\right|$$

where F(x) and G(x) represent the observed cumulative function of the samples m and n, respectively. Then, the *D* statistic is compared with the respective Kolmogorov–Smirnov distribution to obtain the p-value of the test. This study uses the scipy.stats.ks_2samp Python Library to perform all the Two-Sample Kolmogorov–Smirnov calculations.

The data analytics process involves an exploratory searching phase for significant differences between groups to select two classes with a higher chance of reaching higher predictive power in machine learning approaches. The Two-Sample Kolmogorov–Smirnov test allows us to explore various combinations between classes. Additionally, results from a study by Cohen et al. (1996) [1] suggest that age-associate changes in the ability to maintain balance begin mid-life (45 years old). After our exploratory searching phase, and considering our data structure, we recommend splitting our classes at 47 years old. With this split, we found classes over 47 years old with significant differences across SOT conditions that can be used as an input for machine learning algorithms. Therefore, we will apply two approaches to assess this study’s aim. Firstly, to train machine learning models with the selected classes with individuals over 47 years old with a higher chance of higher predictive performance. Secondly, to train machine learning models using all the individuals of our dataset.

## 3. Results

After applying the exploratory searching phase of two classes with a higher chance of reaching higher predictive power by using the Shapiro–Wilk test (normality test) and Two-Sample Kolmogorov–Smirnov test (to compare two samples), the results showed that by splitting the data for younger patients (≤47 years old) and older patients (>47 years old), the classes with significant differences across all the SOT conditions are Normal individuals and TBI patients older than 47 years old; please see Table A2 for more details of the Two-Sample Kolmogorov–Smirnov Test results. The Shapiro–Wilk test showed that these two groups were drawn from a non-normal distribution; please see Table A1 for more information. Additionally, the ANOVA test was applied to the individuals’ weight in these two groups, showing no significant difference. Therefore, Normal individuals and TBI patients over 47 years old were selected to train and test machine learning models in the first approach.

The second approach consists of training and testing machine learning models using the ApEn values from all the patients in the data. It was found that one of the classes has a different weight mean population. Further analysis shows that the Imbalance class (class 1) is the only group significantly different from the others. Based on the fact that the exploratory analysis of the ApEn values for each SOT condition per each class presented similar locations, shapes, and dispersion in their distributions, this study proceeded to train machine learning models to classify four classes of balance impairments (Normal Balance, Imbalance, TBI, and UVW Right).

Our first approach predicts two diagnoses (Normal Balance or TBI) among patients over 47 years old. The second approach predicts four diagnoses for all patients (Normal Balance, Imbalance, TBI, and UVW Right). Table 1 shows the results of the prediction metrics for accuracy, precision, recall, and f1-score. In general, the best f1-score results were found with the model LR, with an f1-score of 76.47%, and SVM, with an f1-score of 76.19%, for the first approach. These are good results considering that only the SOT raw data were used to reach this power of prediction. On the other hand, in the second approach, the best f1-score results were obtained for LR with an f1-score of 32.28%, followed by LDA with an f1-score of 32%. This lower performance was expected since the location and shape of the ApEn for each COP condition distribution were similar.

## 4. Discussion

Detrending COP trajectories is a critical step in pre-processing SOT raw data. An important consideration is that the physiological bases of the COP are not completely well-understood. In that sense, EMD is well-suited to detrend time series such as COP, since it does not rely on a priori signal nature assumptions or on specific bases, such as sinusoidal or Haar wavelet function, to decompose the COP time series into IMFs [20]. The resulting IMFs of the EMD decomposition can be recombined so that the IMF that captures the higher frequencies can be excluded, thus, obtaining a detrended time series.

This study combined the first five IMFs and the resulting detrended COP could then be processed with ApEn. However, the different patient behaviour during each SOT condition generated dissimilar numbers of IMFs. Therefore, recombining a fixed number of IMFs to detrend the COP of all patients could result in information loss in the detrended COP time series. This possibility was verified by testing if the resulting detrended COP came from a random process by shuffling the detrended COP and comparing the ApEn with the non-shuffled detrended COP. Figure 5 shows that the non-shuffled COP ApEn values are lower than the shuffled ones, which means that this methodology still captures dynamics patterns of the postural control of the patient.

The postural control system in humans can be described as a non-linear behaviour [15]. In non-linear dynamics systems, entropy quantifies the regularity of the system. This study used ApEn to indicate how regular or predictable a time series is by measuring its degree of randomness, regardless of the process that generated it. Thus, the more regular or predictable a time series is, the less complex it will be, which is indicative of a less adaptive system [16]. Less adaptive systems are associated with balance disorder impairments; therefore, there are usually represented with lower ApEn values. In our cases, balance disorders that present higher differences among their ApEn across the COP of its SOT conditions are more likely to be correctly classified by machine learning models.

Balance disorder impairments remain a diagnostic challenge and frustrating task for clinicians [3,30,31,32,33]. An appropriate balance disorder diagnosis involves the evaluation of a constellation of symptoms and underlying causes. This study has reached an f1-score up to 76.47% for LR and 76.19% for SVM using only ApEn to characterise each patient. Even though these results are not a definitive final solution to performing diagnosis, we can classify them as good models, considering the current complexity involved in diagnosing balance impairments and the fact that only ApEn was involved in the result.

Previous studies have been using COP, EMP, and ApEn to enhance the current function of the SOT protocol, which evaluates each sensory system’s contribution to maintaining balance. This article aims to take a further step and use the ApEn of the COP trajectories to extend the usability of SOT raw data to diagnose patients. Our result shows that machine learning approaches can extract valuable information from SOT raw data to classify patients with balance impairments. Therefore, there is a potential to expand the usability of SOT raw data to help clinicians further.

There are several ways to improve the performance of machine learning models, by applying methods such as feature engineering, adjusting hyperparameters, and trying multiple algorithms. Future works can focus on two main approaches. On the one hand, since we are using only ApEn in this study, increasing the dimensionality by adding features from clinical historical patient records could be a methodology to improve our current f1-score performance. Expanding the dimensionality and keeping the same number of instances (patients) could lead to overfitting; therefore, performance evaluation should consider a process to evaluate overfitting. On the other hand, the methodology explained in this paper with a large cohort can be used to improve performance. Ideally, future works will have a mix of the two approaches to improve the f1-score of our model, a large cohort, and enrichment of patient information with their historical patient records.

Machine learning methods can create their own rules directly from the data to perform classification tasks. This set of rules is so complex that, most of the time, it is not interpretable by humans. For that reason, they are called the black box of the machine learning model. This study has shown that machine learning can extract useful information from SOT raw data to classify balance impairments. Having said that, future work can focus on developing explainability methods to make the black box (set of internal rules and mechanism of machine learning models) interpretable to humans. This information can help clinicians in their diagnosing process and could help create new lines of research for new findings.

### Limitations

One limitation of this study was related to the access to patients’ clinical records. Clinical record information contains features that could help improve machine learning models’ performance. Patient data privacy policies only allow this study the use of anonymised SOT raw data. Future works can consider expanding access to anonymised clinical records, which could lead to better performance.

Our study shows that Normal individuals and TBI patients have the best chance of reaching better machine-learning performance. However, future studies could consider the evaluation of a larger cohort to generalise this behaviour over Imbalance and UVW Right patients.

Additionally, machine learning models primarily focus on classification rather than showing the underlying personalised features that the model took into account to classify a patient with a particular balance disorder. In healthcare, this type of analysis is essential for the clinician diagnosis process. Extension of this study can consider the development of explainability methods to provide these insights to clinicians.

## 5. Conclusions

The human body is described as a dynamic system with redundancy in the degree of freedom, allowing it to generate numerous strategies to adapt to its environmental conditions. The SOT protocol helps measure the sensory system’s contributions under various conditions; however, it does not provide a final balance disorder diagnosis. This study has described methodological considerations associated with processing COP trajectories with EMD and entropy measures, such as ApEn, that can contribute to inserting information in machine learning models related to the level of adaptability of the patient’s balance system. Only using ApEn values, our models reached f1-scores up to 76.47% for logistic regression (LR) and 76.19% for support vector machine (SVM). The results show that there is a potential to expand the current usage of SOT raw data to help balance disorder diagnosis, and future research could use ApEn conjointly with other clinical patient records data to evaluate machine learning performance improvements.

## Figures and Tables

**Table 1:** Machine learning models—prediction metrics performance.

Models	Accuracy	Precision	Recall	F1 Score
Patients > 47||Normal Balance vs. TBI				
LR	72.74%	72.22%	81.25%	76.47%
RF	72.41%	78.57%	68.75%	73.33%
LDA	62.06%	60.87%	87.50%	71.79%
SVM	65.51%	61.54%	100.00%	76.19%
All Patients || Normal Balance, Imbalance, TBI, UVW Right				
LR	43.69%	36.04%	34.73%	32.28%
RF	40.34%	32.50%	31.28%	30.31%
LDA	42.86%	34.44%	33.85%	32.07%
SVM	39.49%	32.06%	31.29%	29.91%

## Data Availability

The data presented in this study are only available to members of this project. The anonymised data are available to other researchers upon reasonable request to the authors and agreement by the clinic locality.

## Appendix A

**Table A1:** Shapiro-Wilk test—Test of normality.

**COFx—Patients ≤ 47 years old**					**COFx—Patients > 47 years old**				
	**Class 0: N**	**Class 1: I**	**Class 2: T**	**Class 3: U**		**Class 0: N**	**Class 1: I**	**Class 2: T**	**Class 3: U**
**n**	**71**	**64**	**46**	**7**	**n**	**59**	**121**	**57**	**50**
Cond1	0.442	0.0424	0.5162	0.0602	Cond1	0.2637	0.0001	0.0086	0.2465
Cond2	0.0085	0.0172	0.0221	0.5669	Cond2	0.0358	0.0000	0.0000	0.0015
Cond3	0.0288	0.0226	0.0137	0.3386	Cond3	0.0643	0.0000	0.0318	0.0007
Cond4	0.5113	0.0067	0.0001	0.7692	Cond4	0.0002	0.0182	0.0037	0.0002
Cond5	0.9594	0.161	0.0001	0.9073	Cond5	0.7898	0.2995	0.128	0.0333
Cond6	0.1344	0.5797	0.0024	0.2274	Cond6	0.1682	0.0501	0.0252	0.0348
**COFy—Patients ≤ 47 years old**					**COFy—Patients > 47 years old**				
	**Class 0: N**	**Class 1: I**	**Class 2: T**	**Class 3: U**		**Class 0: N**	**Class 1: I**	**Class 2: T**	**Class 3: U**
**n**	**71**	**64**	**46**	**7**	**n**	**59**	**121**	**57**	**50**
Cond1	0.0004	0.0002	0.0048	0.0039	Cond1	0.0016	0.0047	0.0000	0.0001
Cond2	0.0000	0.0000	0.0000	0.1991	Cond2	0.0127	0.0003	0.0000	0.0018
Cond3	0.0000	0.0000	0.0000	0.2693	Cond3	0.0000	0.0001	0.0000	0.0000
Cond4	0.016	0.0051	0.0727	0.9099	Cond4	0.0633	0.242	0.0015	0.7876
Cond5	0.3549	0.098	0.0206	0.6259	Cond5	0.3491	0.0343	0.0197	0.0905
Cond6	0.0726	0.2741	0.1112	0.7791	Cond6	0.1924	0.0192	0.0276	0.0067

Null Hypothesis: Samples come from a normally distributed population. Red colours represent *p*-values < 0.05. Yellow colours represent *p*-values < 0.10.

Table A1 provides information about the normality of the SOT conditions using the Shapiro–Wilk test. According to this table, all the conditions with red numbers (*p*-values < 0.05) represent distributions that are not normal. Additionally, the conditions with yellow numbers (0.05 ≤ *p*-values ≤ 0.10) were close to being rejected as normal distribution.

**Table A2:** Two-Sample Kolmogorov–Smirnov Test.

**COFx—Patients ≤ 47 years old**					**COFx—Patients > 47 years old**				
	**Class 0: N**	**Class 1: I**	**Class 2: T**	**Class 3: U**		**Class 0: N**	**Class 1: I**	**Class 2: T**	**Class 3: U**
**n**	**71**	**64**	**46**	**7**	**n**	**59**	**121**	**57**	**50**
Cond1	0.60 ± 0.23 ^I^	0.71 ± 0.26 ^N,TT^	0.52 ± 0.21 ^II^	0.66 ± 0.27	Cond1	0.60 ± 0.23 ^I,T^	0.55 ± 0.26 ^N^	0.47 ± 0.27 ^N^	0.53 ± 0.23
Cond2	0.52 ± 0.24 ^T^	0.53 ± 0.26 ^T^	0.40 ± 0.19 ^N,I^	0.49 ± 0.21	Cond2	0.47 ± 0.20 ^TT^	0.44 ± 0.24 ^TT^	0.28 ± 0.16 ^NN,II,UU^	0.41 ± 0.18 ^TT^
Cond3	0.58 ± 0.25 ^TT^	0.56±0.28 ^TT^	0.43 ± 0.24 ^NN,II^	0.50 ± 0.25	Cond3	0.51 ± 0.23 ^TT^	0.48 ± 0.25 ^TT^	0.33 ± 0.17 ^NN,II,UU^	0.47 ± 0.20 ^TT^
Cond4	0.42 ± 0.14 ^TT^	0.44 ± 0.23 ^TT^	0.33 ± 0.16 ^NN,II^	0.46 ± 0.23	Cond4	0.42 ± 0.19 ^TT^	0.34 ± 0.15	0.28 ± 0.14 ^NN,U^	0.39 ± 0.17 ^T^
Cond5	0.30 ± 0.10 ^TT^	0.29 ± 0.12	0.25 ± 0.13 ^NN^	0.28 ± 0.09	Cond5	0.30 ± 0.11 ^TT^	0.26 ± 0.12 ^TT^	0.20 ± 0.11 ^NN,II,UU^	0.27 ± 0.14 ^TT^
Cond6	0.40 ± 0.13 ^I,TT^	0.33 ± 0.15 ^N,T^	0.30 ± 0.14 ^NN,I^	0.40 ± 0.14	Cond6	0.32 ± 0.16 ^TT^	0.28 ± 0.14 ^T^	0.24 ± 0.13 ^NN,I,U^	0.29 ± 0.14 ^T^
**COFy—Patients ≤ 47 years old**					**COFy—Patients > 47 years old**				
	**Class 0: N**	**Class 1: I**	**Class 2: T**	**Class 3: U**		**Class 0: N**	**Class 1: I**	**Class 2: T**	**Class 3: U**
**n**	**71**	**64**	**46**	**7**	**n**	**59**	**121**	**57**	**50**
Cond1	0.43 ± 0.23	0.44 ± 0.23	0.37 ± 0.21	0.39 ± 0.29	Cond1	0.42 ± 0.20 ^TT^	0.39 ± 0.19 ^TT^	0.31 ± 0.20 ^NN,II,U^	0.38 ± 0.17 ^T^
Cond2	0.25 ± 0.12	0.23 ± 0.15	0.2 ± 0.12	0.21 ± 0.11	Cond2	0.27 ± 0.12 ^TT^	0.28 ± 0.13 ^TT^	0.17 ± 0.08 ^NN,II,UU^	0.24 ± 0.12 ^TT^
Cond3	0.34 ± 0.16 ^II,TT^	0.28 ± 0.19 ^NN^	0.24 ± 0.13 ^NN^	0.32 ± 0.18	Cond3	0.33 ± 0.15 ^TT^	0.34 ± 0.17 ^TT^	0.24 ± 0.12 ^NN,II,UU^	0.32 ± 0.14 ^TT^
Cond4	0.25 ± 0.11 ^I^	0.21 ± 0.1 ^N^	0.21 ± 0.1	0.22 ± 0.13	Cond4	0.28 ± 0.10 ^TT^	0.28 ± 0.12 ^TT^	0.20 ± 0.12 ^NN,II,UU^	0.27 ± 0.09 ^TT^
Cond5	0.20 ± 0.07	0.19 ± 0.07	0.17 ± 0.09	0.20 ± 0.08	Cond5	0.23 ± 0.09 ^TT^	0.21 ± 0.11	0.18 ± 0.11 ^NN,II^	0.22 ± 0.12
Cond6	0.27 ± 0.10 ^II,TT^	0.22 ± 0.11 ^NN^	0.20 ± 0.1 ^NN^	0.23 ± 0.13	Cond6	0.26 ± 0.12	0.26 ± 0.13	0.23 ± 0.12	0.27 ± 0.12

Mark of “N” means significant difference in comparison with Class 0: Normal Balance. Mark of “I” means significant difference in comparison with Class 1: Imbalance. Mark of “T” means significant difference in comparison with Class 2: TBI. Mark of “U” means significant difference in comparison with Class 3: UVW Right. Single character represents *p*-values < 0.05. Twin characters represents *p*-values < 0.01.

Table A2 provides information about comparing each diagnosis with the other(s) using the Two-Sample Kolmogorov–Smirnov Test. Those diagnoses with a significant difference are represented with an initial letter diagnosis superscript to indicate that they are different. According to Table A2, Normal Balance (N) and Traumatic Brain Injury (T) patients 47 years old or older show a consistent difference across all the SOT conditions.
